# Investigation of acute-phase proteins and cytokines response in goats with contagious caprine pleuropneumonia with special reference to their diagnostic accuracy

**DOI:** 10.7717/peerj.10394

**Published:** 2020-11-17

**Authors:** Wael El-Deeb, Mahmoud Fayez, Ibrahim Elsohaby, Mohamed Salem, Abdulrhman Alhaider, Mahmoud Kandeel

**Affiliations:** 1 Clinical Sciences, College of Veterinary Medicine, King Faisal University, Al-Ahsa, Saudi Arabia; 2 Department of Internal Medicine, Infectious Diseases and Fish Diseases, Faculty of Veterinary Medicine, Mansoura University, Mansoura, Aldakahlia, Egypt; 3 Department of Bacteriology, Veterinary Serum and Vaccine Research Institute, Ministry of Agriculture, Cairo, Egypt; 4 Bacteriology, Al Ahsa Veterinary Diagnostic Laboratory, Alhofof, Al-ahsa, Saudi Arabia; 5 Department of Animal Medicine, Faculty of Veterinary Medicine, Zagazig University, Zagazig, Sharkia, Egypt; 6 Department of Health Management, Atlantic Veterinary College, University of Prince Edward Island, Charlottetown, PEI, Canada; 7 Department of Medicine and Infectious Diseases, Faulty of Veterinary Medicine, Cairo University, Cairo, Egypt; 8 Department of Biomedical Sciences, King Faisal University, Al-Ahsa, Saudi Arabia

**Keywords:** C-reactive protein, Procalcitonin, Haptoglobin, Serum amyloid a, Cytokines

## Abstract

Acute-phase proteins (APPs) have always had valued diagnostic potentialities in response to infection. This study aimed to evaluate the diagnostic accuracy of selected APPs and proinflammatory cytokines (PIC) in goats with contagious caprine pleuropneumonia (CCPP) under field conditions. Moreover, to highlight the role of tested biomarkers in CCPP pathogenesis. Fifty-eight goats (38 confirmed cases with CCPP and 20 healthy controls) were involved in this investigation. C-reactive protein (CRP), procalcitonin (PCT), haptoglobin (HP), fibrinogen (Fb), serum amyloid A (SAA), selected PIC (IL1-*α*, IL1-*β*, IL-6, interferon-gamma (IFN-*γ*) and tumor necrosis factor-alpha (TNF-*α*)) levels were investigated in serum samples from all goats under investigation. Latex agglutination test was used for diagnosis of goats with CCPP. For microbiological investigations, nasopharyngeal swabs (from all goats), lung tissues and pleural fluids (from only necropsied goats) were collected. This study revealed that all tested parameters have a high to moderate degree of diagnostic performance for CCPP. Magnitudes of increase in levels of APPs (CRP, HP and SAA) were stronger than PIC, IFN-*γ*, Fb and PCT. All tested parameters showed high diagnostic accuracy (AUROC >90%), except HP (AUROC = 87.3%) and IFN-*γ* (AUROC = 78.8%) showed moderate accuracy in differentiation of goats with and without CCPP infection. For detecting goats with and without CCPP infection, HP had the lowest sensitivity (Se = 81.6%) and Fb had the lowest specificity (Sp = 85.0%) among the APPs parameters tested. However, PCT showed the highest Se (100%) and Sp (95.0%) to detect goats with and without CCPP infection among tested parameters. Conclusively, this study endorses the significance of selected APPs and PIC as additional screening diagnostic parameters for naturally occurring CCPP in goats. However, it does not replace traditional methods for diagnosis of CCPP in goats. Furthermore, APPs and PIC have an important role in disease pathogenesis in goats.

## Introduction

Respiratory problems are accountable for significant losses among goats and sheep flocks worldwide. Regardless of the causative agent, small ruminants’ respiratory diseases subsidize to 5.6% of this species’ total diseases and cause about 50% of total mortalities (Nicholas, Ayling & McAuliffe, 2008). Contagious caprine pleuropneumonia (CCPP) caused by *Mycoplasma capricolum* subspecies *capripneumoniae* (MCCP) is considered as a contagious and fatal disease of goats listed by the World Organization for Animal Health which leads to high losses in goat flocks (Thiaucourt & Bolske, 1996; Nicholas, Ayling & McAuliffe, 2008; El-Deeb et al., 2017). The loss of diseased goats with the acute form will occur within 7–10 days of infection. However, goats with sub-acute or chronic forms may show mild symptoms, and the severity of the disease tends to be mild. In the chronic form of CCPP, there are chronic cough, nasal discharge, weakness, and loss of condition (Samiullah, 2013; Tharwat & El-Manakhly, 2016; El-Deeb et al., 2017).

Acute-phase response (APR) is a defensive immune response manifested by the secretion of large quantities of acute-phase proteins (APPs) that increased in circulation above their normal levels to control or limits the extent of infection or inflammation. Checking APPs changes revealed a valued diagnostic and prognostic potentiality throughout inflammation and infection (Eckersall, 2000). Nevertheless, there are considerable differences in APR among different species of animals. Some APPs changes have the same way in both goat and sheep, while others display different APR among sheep and goats (Gonzalez et al., 2008).

Haptoglobin (HP) and serum amyloid A (SAA) are measured as major APPs in goats. However, fibrinogen (Fb) contributes as a moderate APP in goat. HP is an *α*2-globulin and has bacteriostatic properties through its capability to bind free hemoglobin (Tirziu, 2009). SAA intercedes phagocytic cells migration to the infection or inflammation site and acts as a chemoattractant (Abdallah et al., 2016). Synthesis of interleukins (ILs) and tumor necrosis factor *α* (TNF-*α*) from the macrophage in response to infection and inflammatory conditions; stimulate the production of APPs from the liver cell (Jain, Gautam & Naseem, 2011). Procalcitonin (PCT) is an APP delivered in the thyroid C cells and is responsible for homeostasis of calcium (a precursor of calcitonin hormone). PCT is measured as a quantifiable and sensitive marker in bacterial infection cases due to its quick increase in circulation after cytokines production (TNF-*α* and IL-6) (Reinhart, Karzai & Meisner, 2000).

Some *Mycoplasma* spp. were previously reported to persuade inflammatory response (through PIC secretion) and numerous immune cell stimulation (Teh, Ho & Williams, 1988). Since *Mycoplasma* spp. lacks a cell wall structure, and the absence of an immune cell stimulator (Staber et al., 1978), the elements accountable for APR’s stimulation have been imprecise for a long time. Quentmeier et al. (1990) was the first report on some *Mycoplasma* spp. and inflammation-inducing factor.

The host immune cells stimulation and production of APR like syntheses of PIC, such as TNF-*α*, interleukins and interferon-*γ* by different phagocytic cells due to *Mycoplasma* infection have been described (Sacchini et al., 2012; Totte et al., 2015) for some but not yet for MCCP. These research findings advocate that the extreme immune responses are persuaded by some *Mycoplasma* spp. play a vital role in the development of pneumonia. Several research studies in cattle, horses and camels were directed to clarify the function of APR in different disease conditions and the role of APPs in laboratory diagnosis (El-Deeb & Iacob, 2012; El-Bahr & El-Deeb, 2013; El-Deeb, Fouda & El-Bahr, 2014; El-Deeb, 2015; El-Deeb & Elmoslemany, 2016; El-Deeb & Buczinski, 2015; El-Deeb et al., 2018; El-Deeb et al., 2020). Contrariwise, a small number of researches were carried out concerning specific viral, parasitic or bacterial infections in goats and sheep (El-Deeb, 2013; El-Deeb & Tharwat, 2015; Haligur & Ozmen, 2011). Moreover, these investigations were concentrated mainly on APPs concentrations in blood or tissues while providing minimal data on their diagnostic performance. Consequently, the objectives of this study were to evaluate the APR in goats with CCPP, assess the role of APPs in disease pathogenesis, and evaluate the diagnostic performance of tested APPs and PIC in goats infected with CCPP.

## Material and Methods

### Study design and animals population

Initially, our investigation was carried out on one goat herd (*n* = 350) located in the eastern region, Saudi Arabia, with a history of respiratory manifestations. All herd animals were not vaccinated with any of Mycoplasma vaccines and reared under intensive production systems. All animals screened for MCCP antibodies by latex agglutination test (Capri LAT). The LAT test is sensitive at the early stage of the disease as long as IgM persists in the serum. Power analysis with a significance level of 5% and 80% power showed that at least 20 goats are needed in each group to identify the significant difference between parameters in a healthy and diseased group, assuming that each parameter concentration increase would only be identified in 5% of the clinically healthy goats. However, an increase in parameter concentrations would be expected in 40% of goats with CCPP.

Serologically positive goats (*n* = 84) were clinically inspected for cardinal signs of CCPP (anorexia, fever, cough, dyspnea, polypnea and nasal discharge). Nasopharyngeal swabs were collected from goats that showed one or more clinical signs (*n* = 84) for microbiological and molecular diagnosis. In addition, lung specimens, pleural fluids and lymph nodes were collected after postmortem examination of recently dead goats (*n* = 8). Accordingly, goats were categorized into two groups as follow: Group 1 (*n* = 20): include goats free from MCCP (clinically healthy and negative to all bacteriological, serological and molecular tests); and Group 2 (*n* = 38): include goats infected by MCCP (one or more of clinical signs were detected and positive only to MCCP by Capri LAT and PCR). The remaining serologically positive goats (*n* = 46) infected by MCCP in combination with others were excluded from the study to overcome misclassification.

Goats from Group 2 (*n* = 38) were treated with one of the following antimicrobials: (tulthromycin (*n* = 17), tetracycline (*n* = 8), danofloxacin (*n* = 13)) and non-steroidal anti-inflammatory drugs (flunixine meglumine (*n* = 24), phenylbutazone (*n* = 14)).

### Sampling

Blood samples were collected from all goats (*n* = 350), and serum was separated by centrifugation and stored at −20 °C to diagnose the MCCP antibody and quantification of selected markers. After proper cleaning of external naris, a nasopharyngeal swab was collected from animals with clinical signs of CCPP using a guarded polyester swab (Culture Swab-Kalayjian, Patterson Veterinary Supply Inc., USA) and sent cooled in transport media to the lab for bacteriological and molecular examination. Lung specimens, pleural fluids and lymph nodes collected from dead goats (*n* = 8) were kept in sterile plastic containers and sent cooled to the lab for bacteriological examination.

### Screening for MCCP antibodies

Serum samples were examined to detect MCCP antibodies using the latex agglutination test (Capri LAT kits, APHA Scientific, Surrey, UK) performed according to the manufacturer’s recommendations.

### Bacteriological examination

Nasopharyngeal swabs, tissue samples and pleural fluids were processed and cultivated for isolation of MCCP according to the recommendations of OIE (OIE, 2014). Mycoplasma isolates were primarily identified based on biochemical and growth inhibition tests, and MCCP was differentiated from other *Mycoplasma mycoides* cluster by specific PCR. For isolating other respiratory pathogens e.g., *Pasteurella multocida* and *Mannheimia haemolytica*, samples were cultivated on sheep blood agar and incubated at 37 °C for 24 h. Suspected colonies were identified biochemically by VITEK 2 Compact (BioMérieux, France).

### Molecular identification of MCCP

DNA was isolated from mycoplasma isolates as well as clinical samples using Qiagen QIAamp DNA mini kit (Qiagen SA, Courtaboeuf, France). PCR was carried out using the MCCP specific primers MCCPF (5′-ATCATTTTTAATCCCTTCAAG-3′) and MCCPR (5′-TACTATGAGTAATTATAATATATGCAA-3′) for an expected amplification product of 316 bp long sequence specific for MCCP according to methods previously described (Woubit et al., 2004, OIE, 2014).

### Determination of APPs and PIC

PCT and CRP levels were analyzed in goat’s serum samples using ELISA test kits (MyBioSource, San Diego, CA, USA). Serum HP was tested in all goats under investigation using non-species specific ELISA kits (Tridelta Development Plc.). Likewise, SAA was analyzed in serum samples using a solid-phase sandwich ELISA (Tridelta Development Plc.). The levels of PIC (IL-1-*α*, IL-1-*β*, and IL-6) and IFN-*γ* were measured from serum samples using commercially available ELISA Kits (CUSABIO Biotech, Wuhan, China). All tests were performed according to each manufacturer’s recommendations.

### Statistical analysis

Descriptive statistics (mean, median and 25th and 75th percentiles) were calculated for each parameter separately in the healthy and CCPP infected goats. The Shapiro–Wilk test was applied, which showed deviation from normality in examined groups. Therefore, the Wilcoxon-Mann–Whitney test was used for non-parametric analysis to evaluate each parameter’s differences in the healthy and CCPP infected goats. The Spearman’s rank correlation coefficients were used to assess the correlation of the parameters.

To assess the diagnostic accuracy of each parameter, receiver operating characteristic (ROC) curves were constructed. Areas under the ROC curve (AUROC) were examined to assess each parameter’s overall accuracy to determine for detection of goats with and without CCPP. An AUROC of 70 to 90% was considered as moderately accurate, an AUROC of >90% was considered highly accurate, and an AUC of 100 would indicate a perfect test (Gardner & Greiner, 2006). In addition, AUROC and Youden index (=maximum [sensitivity + specificity − 1]) were used to identify the optimal cut-off values for the detection of goats with and without CCPP. Diagnostic test characteristics such as sensitivity (Se), specificity (Sp) and accuracy were calculated for each parameter. Furthermore, the level of agreement between goats categorized as healthy or as CCPP positive was evaluated by Cohen’s kappa statistic (*κ*). Results were considered significant at *P*-value < 0.05. The calculations were carried out using Stata statistical software program for Windows (version 16.1; StataCorp, 2019).

## Results

**Figure 1:**
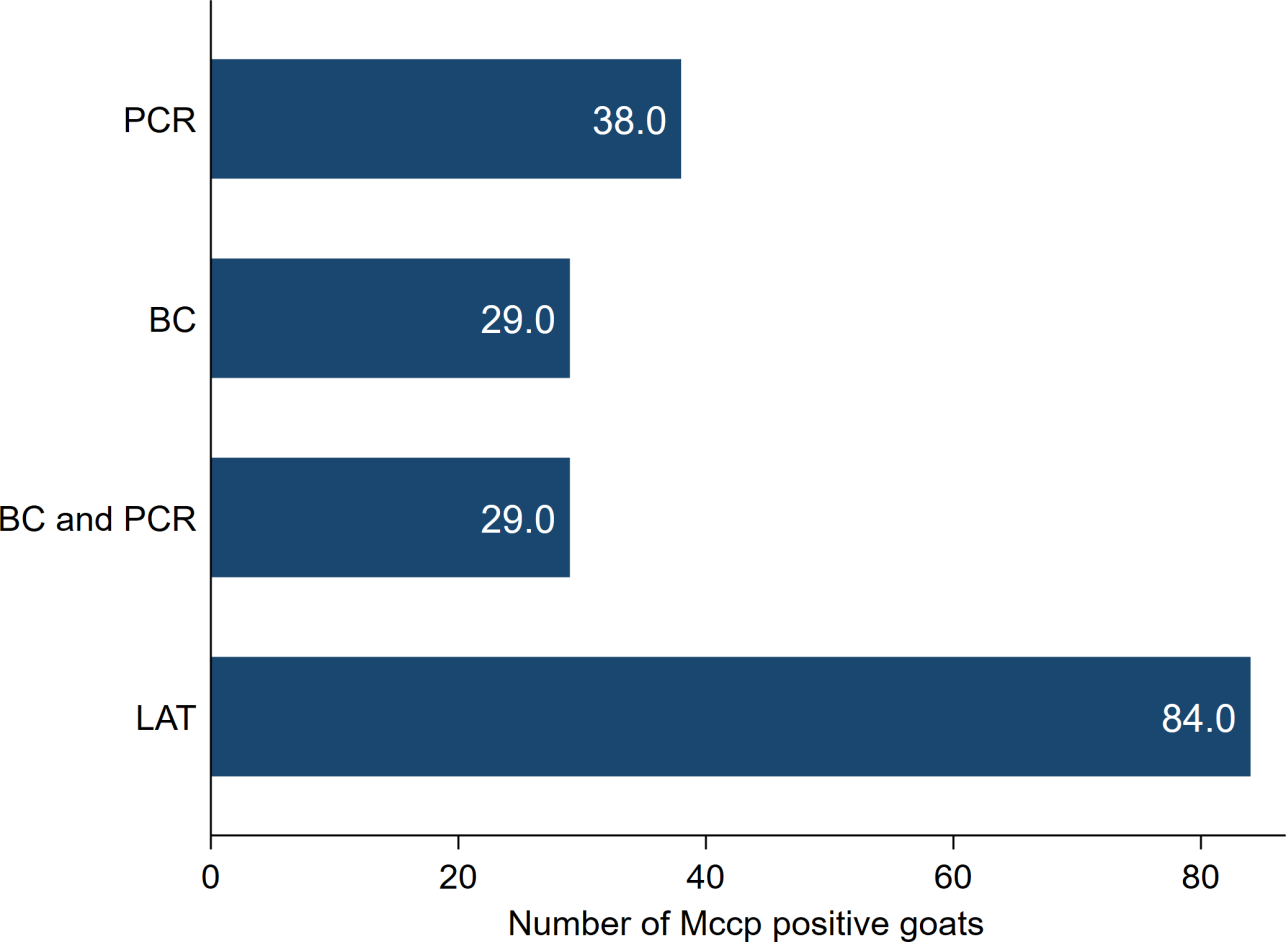
The number of *Mycoplasma capricolum* subspecies *capripneumoniae* (MCCP) positive goats using latex agglutination test (LAT), bacterial culture (BC) and PCR.

Goats with CCPP showed different clinical signs including fever (35/38), severe productive cough (26/38), dyspnea, nasal discharge and extended neck (37/38). The necropsy of goats with CCPP revealed a characteristic fibrinous pleuropneumonia with lung hepatization, adhesion with thoracic wall and fibrinous pleurisy. Moreover, excessive accumulation of straw-colored pleural fluid and granular lung appearance on the cut section were also observed. The number of CCPP positive goats with the different laboratory tests used for diagnosis and confirmation was presented in Fig. 1.

 All 38 goats included in this investigation (Group 2) tested positive with the LAT and were confirmed as CCPP positive animals after applying both PCR and/or bacterial culture on nasopharyngeal swabs tissue samples.

The APPs, PIC, IFN-*γ* and PCT levels were higher in goats with CCPP compared to control healthy ones (Table 1). In addition, magnitudes of increase in levels of APPs (HP, SAA and CRP) were stronger than PIC, IFN-*γ*, Fb and PCT. Spearman’s correlation analysis showed high positive correlations between SAA (*r* = 0.75), PCT (*r* = 0.82) and CRP (*r* = 0.78) in healthy and CCPP infected goats (Table 2). Additionally, moderate positive correlations were found between HP (*r* = 0.62), PIC (*r* = 0.63 to 0.72) and Fb (*r* = 0.71). However, a weak positive correlation was reported between IFN-*γ* (*r* = 0.48) in healthy and CCPP infected goats.

**Table 1:** Descriptive statistics of blood biomarkers in healthy and contagious caprine pleuropneumonia (CCPP) infected goats.

**Parameters**^a^	**Healthy goats (*n* = 20)**				**Goats with CCPP (*n* = 38)**				***P*-value**^b^
	**Mean**	**Median**	**25%**	**75%**	**Mean**	**Median**	**25%**	**75%**	
HP (g/L)	0.093	0.059	0.049	0.062	1.67	1.91	1.63	2.15	<0.0001
SAA (µg/mL)	4.32	4.56	3.96	4.88	27.11	29.36	28.26	31.54	<0.0001
Fb (g/L)	2.34	2.36	2.28	2.41	3.49	3.66	2.51	4.12	<0.0001
CRP (µg/mL)	50.41	50.33	49.34	52.34	110.43	112.61	108.25	120.37	<0.0001
IL1-*α* (pg/mL)	13.48	13.76	12.36	14.75	24.94	26.35	25.36	28.26	<0.0001
IL1-*β* (pg/mL)	18.49	18.53	17.26	19.45	29.30	30.26	29.45	31.45	<0.0001
TNF-*α* (pg/mL)	8.63	8.58	8.05	9.25	17.69	19.25	18.36	20.11	<0.0001
IL6 (pg/mL)	10.63	10.41	9.51	11.41	16.47	17.4	15.45	18.36	<0.0001
IFN-*γ* (pg/mL)	10.02	10.31	9.08	10.80	14.52	16.30	10.36	17.26	0.0002
PCT (pg/mL)	0.499	0.495	0.47	0.53	1.97	1.95	1.78	2.10	<0.0001

**Notes.**

a HP, haptoglobin; SAA, serum amyloid A; Fb, fibrinogen; CRP, C-reactive protein; IL1-*α*, interleukin 1-alpha; IL1-*β*, interleukin 1-beta; TNF-*α*, tumor necrosis factor-alpha; IL6, interleukin 6; IFN-*γ*, Interferon-gamma; PCT, procalcitonin.

b *P* value: resulting from the non-parametric Wilcoxon-Mann-Whitney test.

**Table 2:** Correlation matrix among blood biomarkers in healthy and contagious caprine pleuropneumonia (CCPP) infected goats.

**Parameters**^a^	**Healthy/ diseased**	**HP**	**SAA**	**Fb**	**CRP**	**IL1-*α***	**IL1-*β***	**TNF-*α***	**IL6**	**IFN-*γ***	**PCT**
**HP**	0.62^a^	1.00									
**SAA**	0.75^a^	0.65^a^	1.00								
**Fb**	0.71^a^	0.49^a^	0.55^a^	1.00							
**CRP**	0.78^a^	0.64^a^	0.82^a^	0.59^a^	1.00						
**IL1-*α***	0.68^a^	0.66^a^	0.44^a^	0.48^a^	0.53^a^	1.00					
**IL1-*β***	0.70^a^	0.72^a^	0.54^a^	0.50^a^	0.58^a^	0.68^a^	1.00				
**TNF-*α***	0.72^a^	0.44^a^	0.55^a^	0.56^a^	0.52^a^	0.59^a^	0.48^a^	1.00			
**IL6**	0.65^a^	0.82^a^	0.55^a^	0.46^a^	0.61^a^	0.70^a^	0.78^a^	0.49^a^	1.00		
**IFN-*γ***	0.48^a^	0.34	0.27	0.46^a^	0.29^a^	0.24	0.29	0.33	0.24	1.00	
**PCT**	0.82^a^	0.52^a^	0.68^a^	0.53^a^	0.71^a^	0.60^a^	0.51^a^	0.48^a^	0.55^a^	0.33	1.00

**Notes.**

HP, haptoglobin; SAA, serum amyloid A; Fb, fibrinogen; CRP, C-reactive protein; IL1-*alpha*, interleukin 1-alpha; IL1-*β*, interleukin 1-beta; TNF-*α*, tumor necrosis factor-alpha; IL6, interleukin 6; IFN-*γ*, Interferon-gamma; PCT, procalcitonin.

a Significant correlation at *P* < 0.001.

**Figure 2:**
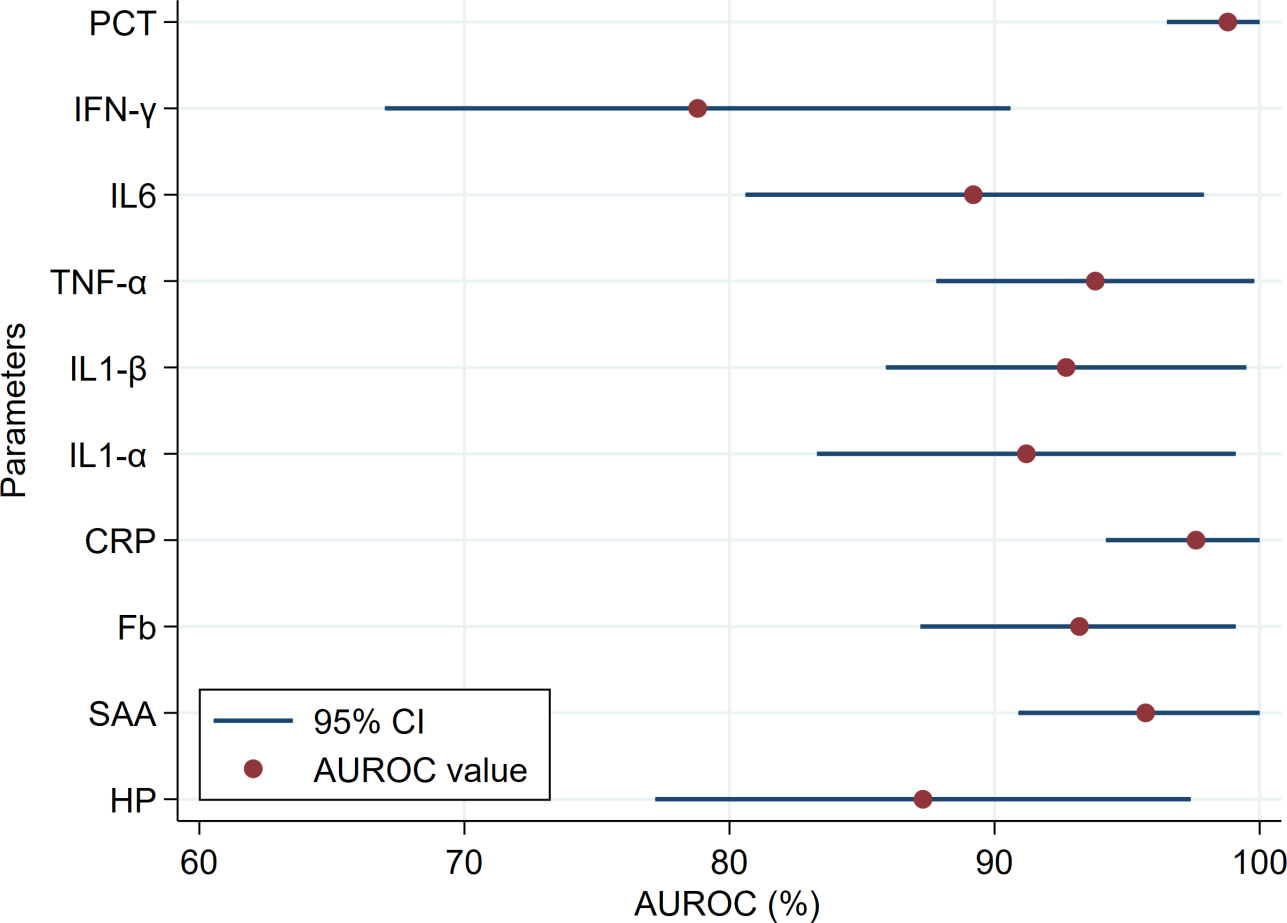
Comparison of the area under the receiver operating characteristic curve (AUROC) and the 95% confidence intervals (CI) of the blood biomarkers in healthy and Contagious Caprine Pleuropneumonia (CCPP) infected goats.

The diagnostic accuracy of APPs, PIC, IFN-*γ* and PCT are presented in Table 3. Overall, APPs showed comparable high diagnostic accuracy (AUROC ranged from 93.2 to 97.6%), except HP showed moderate accuracy (AUROC = 87.3%; Fig. 2). For the detection of goats with and without CCPP infection, HP and Fb had the lowest Se (81.6%) and Sp (85.0%), respectively, among the APPs parameters tested. The diagnostic accuracy of all PIC was high (AUROC ranged from 89.2 to 93.8%). The IFN-*γ* showed a moderate degree of accuracy (AUROC = 78.8%; Fig. 2) and lowest Se (68.4%) for detection of goats with and without CCPP infection (Table 3). However, PCT showed the highest accuracy (AUROC = 98.6%) and highest Se (100%) and Sp (95.0%) for detection of goats with and without CCPP infection among tested parameters.

**Table 3:** Diagnostic test characteristics of blood biomarkers in healthy and contagious caprine pleuropneumonia (CCPP) infected goats.

**Parameters**^a^	**Threshold**	**Diagnostic characteristics (%)**^b^				***J***^c^	*κ*^d^	**CCPP -/+**	**Test -/+**
		**Se (95% CI)**	**Sp (95% CI)**	**Accuracy**	**AUROC (95% CI)**				
HP (g/L)	≥0.54	81.6 (65.7–92.3)	95.0 (75.1–99.9)	86.2	87.3 (77.2–97.4)	0.77	0.72	20/38	26/32
SAA (µg/mL)	≥5.26	86.8 (71.9–95.6)	95.0 (75.1–99.9)	89.7	95.7 (90.9–100)	0.82	0.78	20/38	24/34
Fb (g/L)	≥2.45	86.8 (71.9–95.6)	85.0 (62.1–96.8)	86.2	93.2 (87.2–99.1)	0.72	0.70	20/38	22/36
CRP (µg/mL)	≥53.45	92.1 (78.6–98.3)	95.0 (75.1–99.9)	93.1	97.6 (94.2–100)	0.87	0.85	20/38	22/36
IL1-*α* (pg/mL)	≥16.25	86.8 (71.9–95.6)	95.0 (75.1–99.9)	89.7	91.2 (83.3–99.1)	0.82	0.78	20/38	24/34
IL1-*β* (pg/mL)	≥20.36	86.8 (71.9–95.6)	90.0 (68.3–98.8)	87.9	92.7 (85.9–99.4)	0.77	0.74	20/38	23/35
TNF-*α* (pg/mL)	≥10.24	84.2 (68.7–94.0)	100 (83.2–100)	89.7	93.8 (87.8–99.8)	0.84	0.79	20/38	26/32
IL-6 (pg/mL)	≥13.26	84.2 (68.7–94.0)	95.0 (75.1–99.9)	87.9	89.2 (80.6–97.9)	0.79	0.75	20/38	25/33
IFN-*γ* (pg/mL)	≥11.36	68.4 (51.3–82.5)	95.0 (75.1–99.9)	77.6	78.8 (66.9–90.6)	0.63	0.56	20/38	27/31
PCT (pg/mL)	≥0.55	100 (90.7–100)	95.0 (75.1–99.9)	98.3	98.6 (96.5–100)	0.95	0.96	20/38	19/39

**Notes.**

a HP, haptoglobin; SAA, serum amyloid A; Fb, fibrinogen; CRP, C-reactive protein; IL1-*α*, interleukin 1-alpha; IL1-*β*, interleukin 1-beta; TNF-*α*, tumor necrosis factor-alpha; IL6, interleukin 6; IFN-*γ*, Interferon-gamma; PCT, procalcitonin.

b Se, sensitivity; Sp, specificity; accuracy, percentage of correctly classified samples; AUROC, area under the receiver operating characteristic curve.

c J, Youden index.

d *κ*, Cohen’s kappa value.

## Discussion

The data of our study is considered the first to screen the levels of PIC and APPs in CCPP cases under field conditions. Furthermore, the obtained data highlights the role of examined APPs and PIC in CCPP pathogenesis in goats. Generally, the data on the association between APPs and clinically occurring CCPP in goats is limited. The antigens of *Mycoplasma,* including polysaccharides, galactan, and lipoprotein showed to activate the goat immune response and trigger the emission of PIC (Chambaud, Wroblewski & Blanchard, 1999; Maritim et al., 2018), which consequently causing pathogenic alterations and hence widespread serofibrinous inflammatory response and fluid exudation especially in lungs, pleura, thorax attachments, and sometimes heart, liver, and kidneys.

CRP is measured as a non-specific blood marker; its serum level is valuable in evaluating the inflammatory process (Pepys & Hirschfield, 2003). In this study, we considered that the elevated CRP levels in goats with CCPP might be important in monitoring the APR of goats against the disease. Moreover, CRP showed good Se (92.1%) for discrimination of cases with CCPP from control healthy ones and could be used as an additional screening diagnostic tool for CCPP in goats. Generally, CRP binds to different bacteria and increases their opsonization, earlier to the secretion of immunoglobulin specific to existent infection (Du Clos & Mold, 2004). Moreover, CRP can detoxify blood toxic substances that arise from damaged tissues that existed clearly in lung tissues of diseased goats (Cermak et al., 1993; Sierra et al., 2004) and possesses good anti-inflammatory properties (Zouki et al., 1997). Comparable to our results, a previous study has detected an increase in CRP in *Mycoplasma pneumoniae* infection (Neeser et al., 2019) after trauma, inflammation, and tissue damage, especially in infections with bacteria (Povoa et al., 1998).

PCT, which is considered a new diagnostic biomarker, was used to identify inflammatory reactions caused by CCPP and characterize diseased goats’ immune response. The secretion of PCT is stimulated by endotoxins or inflammatory mediators created in reaction to bacterial infections. Therefore, it may be valuable in distinguishing viral and bacterial infections (Joo et al., 2011; Schuetz et al., 2011). Likewise, PCT is used to diagnose sepsis in human medicine (Schuetz et al., 2011). It has been stated that PCT is a predictor of inflammatory immune response parameters like CRP, body temperature, and leukocyte profile. In this study, PCT showed the highest accuracy for detection of goats with and without MCCP infection among the tested parameters. PCT was previously used for the diagnosis of many inflammatory diseases and to evaluate the efficacy of treatment protocol (El-Deeb et al., 2020). While PCT is recognized as a marker of bacterial infections, it is also elevated in *Mycoplasma pneumoniae* infection (Neeser et al., 2019), acute malaria, fungal (Gunal & Barut, 2009) and bacterial infections (El-Deeb et al., 2020).

In ruminants, HP acts as the main APP produced mainly by hepatocytes. It could be perceived in subclinical infection (Godson et al., 1996). In our study, goats with CCPP showed significantly elevated HP levels compared with healthy goats demonstrating a strong APR to the MCCP infection. Higher serum HP levels could be persuaded by tissue injury (pleuropneumonia in this study) following inflammation and/or infection (Blackmore, 1988). It was previously reported that HP has significant bacteriostatic potentialities by binding of free hemoglobin (Hb) with subsequent formation of HP-Hb complexes (removed by the action of the reticuloendothelial system), precluding iron needed for the growth of bacteria (Eaton et al., 1982; Jain, Gautam & Naseem, 2011). In this study, HP showed moderate diagnostic accuracy for goats with CCPP and the lowest sensitivity among the APPs parameters tested. Our findings agree with preceding researches on the bovine respiratory disease (Nikunen et al., 2007; El-Deeb et al., 2020). Comparable findings were perceived in experimental pneumonia in sheep (Pfeffer & Rogers, 1989), ruminal acidosis in goats (Gonzalez et al., 2010) and do with pregnancy toxemia (Trevisi et al., 2005; Gonzalez et al., 2011). On the other side, other research carried on feedlots revealed an inadequate relationship between HP and clinical respiratory tract disorders (Young et al., 1996).

Our data showed that levels of SAA elevated significantly in goats with CCPP when compared with control goats. Moreover, in this study, SAA showed good diagnostic accuracy for CCPP cases. Higher SAA could be accredited to its role in transporting, binding, and scavenging lipoproteins from bacteria and damaged cells during the inflammatory process (Pannen & Robotham, 1995). Additionally, SAA is essential for the APR through activation of phagocytic cells (macrophage and neutrophil) or coliform bacteria elimination (Urieli-Shoval, Linke & Matzner, 2000; Murata, Shimada & Yoshioka, 2004; Orro et al., 2011). Likewise, this protein has the affinity to bind Gram-negative bacteria and the stimulation of phagocytosis process (Larson et al., 2005). Higher SAA levels were formerly detected in calves with respiratory problems (Horadagoda et al., 1994; Nikunen et al., 2007; Orro et al., 2011; El-Deeb et al., 2020). While some investigations proposed that SAA is a more specific parameter for diagnosis of infection with the bovine respiratory syncytial virus in cattle (Heegaard et al., 2000) and acute inflammation in calves with *Pasteurella haemolytica* infection (Horadagoda et al., 1994).

In small ruminants, Fb is responsible for blood clotting and tissue repair described by a minor immune response during inflammation or infection (Hirvonen, Hietakorpi & Saloniemi, 1997). Moreover, in inflammatory conditions and bacterial infection, Fb is measured as a reliable diagnostic parameter for many diseases (Pfeffer et al., 1993; Nikunen et al., 2007; Gonzalez et al., 2008). This study revealed that Fb levels in goats with CCPP were significantly elevated when compared with control ones. The high serum Fb levels may be endorsed to Fb’s role in tissue repairing, inflammatory immune response, and controlling hemostasis (Feldman et al., 2000). In this study, Fb had the lowest specificity among examined APPs to detect goats with and without CCPP infection.

The PIC are the main stimulators of APR and for the production of APPs (Baumann & Gauldie, 1994). Consequently, in our study, the high APPs and strong APR levels were attributed to the secretion of different kinds of PIC. Lipoproteins in *Mycoplasma* were previously identified as an inflammation-inducing parameter through PIC’s induction (Quentmeier et al., 1990). Yang et al. (2002) first stated the connection between cytoadherence and the stimulation of APR. Mycoplasma-Lipoproteins stimulate APR through Toll-like receptors (TLR) 2 (Shimizu et al., 2014). Moreover, the *Mycoplasma* cytoadherence in the respiratory tracts stimulates APR through inflammasome (an intracellular receptor protein complex) (Yang et al., 2002; Shimizu et al., 2014).

Furthermore, *Mycoplasma* stimulates ATP efflux from the host cells. ATP efflux activated inflammasomes through the P2X7 receptor, which is succeeded by releasing IL-1*β* into the circulation (detected at high levels in our study) (Shimizu et al., 2011). In the same concern, Sugiyama et al. (2015) reported that *Mycoplasma* stimulates IL-1*β* in a dendritic cell line. Comparable to our results, *Mycoplasma mycoides* subsp. *capri* (heat-killed suspensions of the closely related pathogens) activated macrophages as well as bone marrow cells and triggered the synthesis of PIC (Rosendal, Levisohn & Gallily, 1995). Similarly, *Mycoplasma mycoides* subsp. *mycoides* strains induced the release of TNF-*α* in macrophages of cattle (Jungi et al., 1996).

In our study, we anticipated an association between higher serum levels of TNF-*α* and CCPP clinical signs, as TNF-*α* is a crucial PIC that it is secreted in response to infection with bacteria and mycoplasmas in large quantities and is accountable for many systemic disease complications (Murphy, Travers & Walport, 2007). Higher serum levels of TNF-*α* were detected in most of the goats following MCCP infection, which confirm the assumption that TNF-*α* mediates inflammatory response in CCPP in goats. Consequently, the higher serum levels could be considered a sign of what is happening in some areas of the goat’s lung with MCCP infection.

IFN-*γ* has been suggested as a valued parameter required for cell protection (Dedieu et al., 2005), as their blood levels were inversely associated with the severity of the disease. Although TNF-*α* and IFN-*γ* may have a vital cell-protective property or initiate protective immune responses in goats with CCPP, their blood levels may be linked to the severity of the MCCP infection amount of mycoplasma in the host.

Van Reeth & Nauwynck (2000) reported comparable results in respiratory troubles in pigs. Our findings are also in harmony with those reported in calves (Pace et al., 1993; Horadagoda et al., 1994; Morsey et al., 1999; El-Deeb et al., 2020) and cattle (Kasimanickam et al., 2013; El-Deeb & Elmoslemany, 2016) with a different bacterial infection. Additionally, Malazdrewich et al. (2001) reported that IL1-*α* and TNF-*α* were elevated in inflammation of respiratory airways.

In this study, the tested biomarkers’ capability to differentiate CCPP cases from healthy goats was evaluated with ROC analysis. Interestingly, APPs showed comparable high diagnostic accuracy, except HP showed moderate accuracy for CCPP cases based on guidelines specified by Swets (1988) and under this study’s conditions. One of the present study limitations is the lack of evaluation of APR after treatment. Future research studies should be focused on the role of PIC and APPs as a guide for treatment protocol.

## Conclusions

The goat immune system activation by MCCP infection is elucidated in this study. Our data show that CCPP caused by MCCP in goats was allied with a significant increase in PIC and APPs, with the highest increase being perceived in CRP, PCT, SAA and HP. Moreover, there was a moderate to high correlation between disease status and APPs and PIC levels. PCT showed the highest accuracy (AUROC = 98.6%) and highest Se (100%) and Sp (95.0%) for detection of goats with and without CCPP infection among the tested parameters. Lastly, this study highlights the value of examined APPs and PIC in CCPP pathogenesis.

##  Supplemental Information

10.7717/peerj.10394/supp-1 Data S1 Raw data

## Additional Information and Declarations

### Field Study Permissions

The following information was supplied relating to field study approvals (i.e., approving body and any reference numbers): Deanship of Scientific Research at King Faisal University Project number: IFT20127.

### Data Availability

The following information was supplied regarding data availability: The raw data is available as Supplemental File.
